# Nanoformulation of valsartan-loaded tablet attenuates L-NAME-induced hypertension: role of Nrf2/PPARγ/AT1 signaling pathway

**DOI:** 10.1007/s00210-025-03993-4

**Published:** 2025-03-26

**Authors:** Hanan Elimam, Khalid M. El-Say, Tarek A. Ahmed, Sylvie Marleau, Zakaria El-Khayat, Mona El-Banna, Jihan Hussein

**Affiliations:** 1 Department of Biochemistry, Faculty of Pharmacy, University of Sadat City, Sadat City, 32897 Egypt; 2 Department of Pharmaceutics and Industrial Pharmacy, Faculty of Pharmacy, Al-Azhar University, Cairo, Egypt; 3 Faculty of Pharmacy, Université de Montréal, Montréal, QC Canada; 4 Department of Medical Biochemistry, National Research Centre, Giza, Egypt

**Keywords:** Hypertension, Valsartan, SNEDS, L-NAME

## Abstract

Hypertension is the most common entity globally, marked by high prevalence and heterogeneous pathophysiology. Oxidative stress is a crucial area of investigation among potential etiologies. We examined the hypothesis that blocking the angiotensin type 1 (AT1) receptor with valsartan (VST) in self-nanoemulsifying delivery systems (SNEDS) and loads in liquisolid tablets (LST-1) or valsartan and hydrochlorothiazide (VST/HCTZ) in SNEDS and loads in liquisolid tablets (LST-2) in comparison with non-SNEDS liquisolid tablets (DCT-3 and DCT-4) would lead to an improvement in hypertension management. The present study aims to explore the molecular mechanisms underlying their effect in N(G)-nitro-L-arginine methyl ester (L-NAME)-induced hypertensive rats. Male Sprague–Dawley rats were given L-NAME (40 mg/kg/day) orally for three weeks to inhibit the endogenous synthesis of nitric oxide (NO). Concurrent treatment with VST or VST/HCTZ liquisolid tablets (20 mg/kg/day for three weeks) resulted in lowering blood pressure (BP), reversing the L-NAME-induced serum NO suppression, enhancing lipid profile, and improving oxidative status. The antioxidant defense of paraoxonase was significantly increased in the LST-1- and LST-2-treated rats compared to the L-NAME-treated rats by 135% and 90%, respectively. Furthermore, SNEDS-loaded VST or SNEDS-loaded VST/HCTZ liquisolid tablets significantly lowered the elevated level of AT1 (*P* < 0.05), showed a marked Nrf2 expression (*P* < 0.01) and overexpressed PPARγ (*P* < 0.05), and suppressed iNOS expression (*P* < 0.0001). These results highlight the remarkable benefits of the novel formula, “SNEDS-loaded VST and SNEDS-loaded VST/HCTZ,” as an alternative therapy in treating hypertension and its complications.

**Graphical abstract:**

Schematic diagram showing the mechanism of SNEDS-loaded VST and SNEDS-loaded VST/HCTZ as potential treatment strategies for hypertension. This mechanism includes the reduction of iNOS expression, antioxidant activity, and AT1R normalization action through activation of the Nrf2/PPARγ signaling cascade.

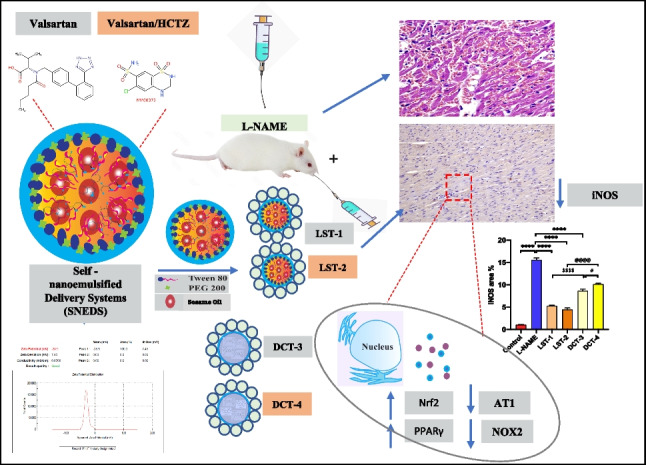

## Introduction

Currently, about 1.28 billion adults aged 30–79 years worldwide have hypertension (blood pressure (BP) ≥ 140/90 mmHg) (Kario 2023). Hypertension significantly contributes to global mortality and morbidity, with rising incidence rates. It has become a global health and financial concern (Fuchs and Whelton 2020; Joseph et al. 2017). Hypertension-related complications, including abnormal renal dynamics, myocardial infarction, coronary heart disease, stroke, and heart failure, damage the cardiovascular system. The most common mechanism involved in the pathophysiology of hypertension is the renin–angiotensin–aldosterone system (RAAS). Several studies have indicated that angiotensin-converting enzyme inhibitors (ACEI) and angiotensin II type 1 receptor blockers (ARBs), two inhibitors of the RAAS, are useful in protecting the cardiovascular system from hypertensive injuries by preventing tissue angiotensin II (Ang II) (Dzau 2005; Ruilope et al. 2005). High BP can cause kidney failure, which is a progressive condition that can take years to manifest (Kim 2023). BP control may help reduce the development of renal failure (Pugh et al. 2019). Studies have demonstrated that ACEI or ARBs significantly reduce proteinuria and delay the progression of renal disease compared to placebo treatment (Silvarino et al. 2019). Furthermore, studies have demonstrated that these medications lower BP, shield hypertensive patients from cardiovascular events, and lessen ventricular remodeling in coronary artery disease patients (Bisognano et al. 2007).

Antihypertensive therapy effectively regulates BP and mitigates cardiovascular events. Valsartan (VST) is an ARB that selectively inhibits Ang II binding to its receptor (Black et al. 2009). VST’s effectiveness, safety, and tolerability have been demonstrated in large-scale heart failure, hypertension, and myocardial infarction studies (Black et al. 2009). Unlike ACEIs, ARBs, including VST, exhibit excellent tolerance and do not induce a dry cough (Baracco and Kapur 2011). Furthermore, VST is advantageous for glucose metabolism and significantly lowers LDL and total cholesterol levels while concurrently lowering blood pressure (Hanefeld and Abletshauser 2001). High-dose VST significantly ameliorated hypertension and decreased the mRNA levels of caspase-3 and transforming growth factor beta-1 (Akashiba et al. 2008). Alternatively, hydrochlorothiazide (HCTZ) was authorized by the FDA of the United States as a drug for the treatment of peripheral edema and hypertension. The thiazide-type diuretic HCTZ prevents salt resorption in the kidney’s distal convoluted tubules. By directly blocking the sodium chloride cotransporter, thiazides have been a dependable class of antihypertensive diuretics for more than 60 years (Ernst and Fravel 2022). Although loop diuretics are usually the first-choice treatment for peripheral edema, HCTZ is an adjuvant therapy (Sica et al. 2011). Beyond the benefits of arterial pressure reduction alone, the VST/HCTZ combination improves blood pressure control efficacy and positively affects target organs, including the kidney and heart (Bains and Smith 2011). The low oral bioavailability of both drugs was caused by their insufficient solubility in water, either alone or in combination. Self-nanoemulsifying delivery systems (SNEDS) are a novel strategy being investigated to solve issues with oral medication delivery. An oil phase, a surfactant, and a cosurfactant, or cosolvent, make up SNEDS. The selection of SNEDS excipients is influenced by several criteria, including SNEDS properties, drug-dissolving capacities, and in vivo considerations (Buya et al. 2020; Nekkanti et al. 2016).

Rats given daily oral doses of N(G)-nitro-L-arginine methyl ester (L-NAME) develop hypertension, heart and kidney hypertrophy, and increased plasma lipid peroxidation and, in addition, plasma nitrite and glutathione concentrations are markedly decreased (Sung et al. 2013). Consequently, this research aims to evaluate the effectiveness of VST alone or in combination with HCTZ in a non-SNEDS formula by contrasting it with their SNEDS formula. Along with their antihypertensive effectiveness, a special consideration for their potential effects on the heart and oxidative stress was explored by measuring a number of gene expression profiles, e.g., angiotensin-1 (AT1), angiotensin-2 (AT2), ACE1, and ACE2, as well as inflammatory/anti-inflammatory mediators, e.g., nuclear factor kappa B (NF-κB), interleukin-6 (IL-6), nuclear factor erythroid 2-related factor 2 (Nrf2), and other biomarkers incovolved in the pathophysiology of hypertension, dyslipidemia, impaired glucose metabolism, and oxidative stress.

The investigation of PPAR agonists in conjunction with other cardiovascular agents, including diuretics and Ang II receptor blockers, has been suggested. Hence, in the present study, we aimed to ameliorate the bioavailability of VST and test it as an agonist for PPAR as a single treatment and in combination with the diuretic HCTZ. The range of PPARγ agonist therapeutic effects will likely result in novel cardiovascular and other disease approaches. The second aim of the present research is to explore the molecular mechanisms underlying their action in L-NAME-induced hypertensive rats. The current research evaluated the modulatory effects of VST and VST/HCTZ on the Nrf2, PPARγ/NF-κB, p38 MAPK, and ERK42/44 trajectories in the heart of the L-NAME-induced hypertensive rat model.

## Materials and methods

### Materials

VST was donated from SAJA Pharmaceutical Co. Ltd., (Jeddah, Saudi Arabia). Sesame oil was obtained from a local vendor (Jeddah, Saudi Arabia). Polyethylene glycol 200 (PEG 200) was purchased from Merck Schuchardt, Hohenbrunn, Germany, and Tween® 80 from Merck KGaA, Darmstadt, Germany. L-NAME was purchased from Sigma Aldrich, Darmstadt, Germany. Ribozol™ RNA Extraction Reagent from VWR International (Radnor, PA, USA) with the PureLink™ RNA Micro-Kit from Invitrogen, Waltham, MA, USA. QuantiFast SYBR Green PCR kit was sourced from Qiagen, Hilden, Germany. The reverse transcription system was acquired from Thermo Scientific, Meridian Rd, Rockford, IL, USA. DTNB, trichloroacetic acid, thiobarbituric acid, and other reagents utilized in the study were procured from Sigma Chemical Company, St. Louis, MO, USA. Rat IL-6 ELISA Kit (Cat# SL0411Ra) and Rat NF-kB ELISA Kit (Cat# SL0537Ra) were purchased from Sunlong Biotech Co., Ltd, China. Magnesium aluminometasilicate (Neusilin®US2) was acquired from Fuji Chemical Industries Co. Ltd., Toyama, Japan. Fumed silica anhydride was procured from Sigma-Aldrich, St. Louis, MO, USA. Talc powder from Whittaker Clark & Daniels, South Plainfield, NJ, USA. Magnesium stearate was supplied by Winlab Laboratory Chemicals Reagents, Leicestershire, UK. Croscarmellose sodium (Ac-Di-Sol®) was supplied by Biosynth International, Inc., San Diego, CA, USA. Methocel (hydroxypropyl methylcellulose) E5 Premium LV (HPMC E5) was acquired from Dow Chemical Company, Midland, USA. Polyclonal anti-iNOS antibody (Cat # GTX130246) was purchased from GeneTex, Irvine, CA, USA.

### Development of SNEDS-loaded liquisolid tablets

#### Formulation and characterization of VST- and HCTZ-loaded SNEDS

The study involved formulating and characterizing SNEDS loaded with VST and HCTZ. The SNEDS consisted of sesame oil, Tween 80, and PEG 400. This formulation was optimized in the previous work for minimal globule size and maximum drug loading (El-Say et al. 2023). The formulation was visually evaluated for appearance and spontaneous emulsification and tested for phase separation and thermodynamic stability through freeze–thaw cycles and centrifugation (Elimam et al. 2022). Particle size, zeta potential, and polydispersity index (PDI) were measured using a Malvern Zetasizer Nano ZSP, with results reported as average values with standard deviations (Aldawsari 2018; Khalid et al. 2017).

#### Preparation of SNEDS-loaded VST and HCTZ liquisolid tablets

Liquisolid tablets (LST) were prepared using the direct compression method as per the USP Pharmacopeia (El-Say et al. 2020). Neusilin® US2 was used as a carrier, blended with SNEDS-loaded VST or SNEDS-loaded VST/HCTZ to ensure even distribution. Fumed silica was added as a coating material until the mixture appeared dry. Methocel® (binder) and Ac-Di-Sol® (superdisintegrant) were then incorporated, followed by sieving. Magnesium stearate (glidant) and talc (lubricant) were added last. The powder blend was assessed for flowability before compression into tablets containing 20 mg of VST alone or 20 mg VST with 3.125 mg HCTZ, using a 9-mm double-punch tablet press. We prepared directly compressed tablets (DCT) without SNEDS for comparison using the same components. Table 1 provides details of all formulations.

**Table 1:** Formulation composition of the prepared tablet formulations

Formula code	Neusilin	Fumed silica	SNEDS	VST	HCTZ	Methocel	Ac-Di-Sol	Talc	Magnesium stearate	Tablet weight
	(mg)									
LST-1	100	20	100	20	–	20	20.8	2.8	2.8	286.4
LST-2	100	20	100	20	3.125	20	20.8	2.8	2.8	289.525
DCT-3	100	20	–	20	–	20	20.8	2.8	2.8	186.4
DCT-4	100	20	–	20	3.125	20	20.8	2.8	2.8	189.525

#### Evaluation of the prepared tablet formulations

The flow behavior of the powder blends was assessed before compression using the Hausner ratio, Carr’s index, and angle of repose according to pH. Eur. standards. Bulk and tapped densities were measured to calculate the Hausner ratio and Carr’s index, indicating the flow behavior of the blends (Hausner 1967).

Mechanical strength was evaluated by inspecting the tablets for defects, testing friability (weight loss after rotation), and measuring hardness using standardized equipment according to the USP pharmacopeial criteria.

Weight variation and content uniformity were assessed by weighing ten tablets from each batch, ensuring consistency in drug content through spectrophotometric analysis at specific wavelengths for VST and HCTZ. Also, in vitro disintegration studies were conducted using a disintegration tester with distilled water at 37 °C ± 0.5 °C, recording the time for complete disintegration without residue. Results were reported as mean and standard deviation.

### In vivo experiments

#### Animals

Male adult Sprague–Dawley rats weighing 160–180 g were acquired from the National Research Center’s animal house. Every animal was housed in a 12-h light/dark cycle with a consistent temperature. The University of Sadat City’s animal house gave them typical laboratory food and unlimited water. The study design and animal treatment adhered to the authorized protocol # RERC-FOP-USC-24–02–03 and the rules of the University of Sadat City’s ethics committee in Sadat City, Egypt, and all procedures complied with the ethical care criteria.

#### Study design

Forty-eight male Sprague–Dawley rats were randomly assigned to six groups (8 rats each): group I (control) were rats treated with normal saline; group II (L-NAME, hypertension model) were rats treated daily with 40 mg/kg of L-NAME orally; group III (LST-1, SNEDS-loaded VST-LST) were hypertensive rats treated with 20 mg/kg/day of LST-1; group IV (LST-2, SNEDS-loaded VST/HCTZ-LST) were hypertensive rats treated with 20 mg/kg/day of LST-2; group V (DCT-3, VST-DCT) were hypertensive rats treated daily with 20 mg/kg of DCT-3; and group VI (DCT-4, VST/HCTZ-DCT) were hypertensive rats treated with 20 mg/kg/day of DCT-4. Based on previous preclinical studies, this dosage of VST was chosen since it was within the range of its therapeutic effects (Liu et al. 2019; Wilkinson-Berka et al. 2001). All rats were treated for three weeks (Fig. 2A).

#### Blood pressure measurement

Conscious rats had their heart rates and systolic and diastolic BP monitored at the beginning of the experiment and every week after that. Before employing the tail-cuff methodology to assess BP, the animals were accommodated in the tubes for a duration ranging from 10 to 20 min each day for five days. The animals were placed into a temperature-regulated heating chamber (Ugo Basile, Italy) for 30 min at 28 °C. Subsequently, the tail was passed through a cuff and a tail-cuff sensor, which was linked to an amplifier (ML 125 NIBP, AD Instruments, Australia). The elevated pulse was registered while the cuff underwent automatic inflation and deflation. The formula below was utilized to determine the arterial BP (Meaney et al. 2000): 

$$\mathrm{ABP}=\mathrm{DBP}+0.412\;(\mathrm{SBP}-\mathrm{DBP})$$

BP, DBP, and SBP are BP, diastolic, and systolic, respectively.

#### Electrocardiography (ECG)

Animals were anesthetized by 45 mg/kg thiopental intraperitoneal injection (Kushikata et al. 2003). The electrocrdiography (ECG) of rats was recorded for 5 min using the ECG PowerLab module, which consisted of PowerLab/8sp, Animal Bio-Amplifier, and Lab Chart 7 software, to analyze the ECG (Abdel-Rahman et al. 2017).

#### Blood and tissue sampling

Rats were given a 12-h fast at the end of the treatment period, and while they were under moderate anesthesia, blood was drawn from the retro-orbital venous plexus. After letting the blood coagulate at 37 °C, it is centrifuged for 15 min at 4000 rpm and 4 °C. After being separated, the serum was kept at 20 °C for biochemical analysis. The rats were euthanized by beheading them; the heart was removed right away and then perfused with ice-cold PBS (pH 7.4) to remove any remaining blood. The aorta was divided into tiny pieces, homogenized in 2 mL of PBS (pH 7.4), centrifuged at 4000 rpm for 15 min at 4 °C, and the supernatant was collected for the biochemical investigation of the left ventricle. The residual part of the heart was utilized for Western blot analysis.

### Histopathology

For histological analysis, the heart specimens were placed in 10% neutral buffered formalin. Paraffin-embedded tissues were sliced to the required thickness (4 μm) using a microtome and subsequently stained with hematoxylin and eosin (H&E) according to the Bancroft method (Bancroft and Gamble 2008) and observed by light microscopy.

### Immunohistochemical analysis

Immunohistochemistry assessed nuclear inducible NOS (iNOS) expression in deparaffinized slices of formalin-fixed hearts utilizing pre-formed paraffin tissue blocks. Then, 4-µm sections were cut into positively charged slides for the immune staining protocol. The heat-induced epitope retrieval step was followed by blocking for endogenous peroxidases using hydrogen peroxide, as detailed previously (Ogaly et al. 2015). Slices were incubated for 2 h at 4 °C in a humidified chamber with rabbit polyclonal anti-iNOS antibody (GeneTex, CA, USA). The tissue slices were incubated for color development with a secondary HRP-labeled detection kit (Bio SB, CA, USA), which was used as per manufacturer instructions. Negative control slides were generated by omitting the primary antibody incubation step. Positive expression was quantified as area percent. Tissue slides were examined with a Leica digital microscope DM4B (Leica, Wetzlar, Germany), and images were taken using a Leica DMC 4500 digital camera linked to LAS-X software (Leica, Wetzlar, Germany). Ten fields per slide were used to get the % positive stained area (Malatinsky et al. 1990).

### Biochemical analysis

#### Determination of serum ALT, AST, and tissue oxidants/antioxidants

Serum alanine aminotransferase (ALT) and aspartate aminotransferase (AST) were measured by utilizing colorimetric kits (BioMed Diagnostics) based on the method of Bergmeyer (Bergmeyer 2012). Malondialdehyde (MDA) and NO were estimated in aorta homogenates according to Ruiz-Larrea et al. (Ruiz-Larrea et al. 1994) and Moshage et al. (Moshage et al. 1995), respectively. Paraoxonase activity was quantified spectrophotometrically utilizing phenylacetate as the substrate. This assay involves arylesterase/paraoxonase catalyzing the hydrolysis of phenyl acetate, yielding phenol. The phenol production rate is assessed by observing the increase in absorbance at 270 nm at 25 °C. The working reagent comprised a 20 mM Tris/HCl buffer at pH 8.0, supplemented with 1 mM CaCl_2_ and 4 mM phenyl acetate as the substrate. Aorta homogenates, diluted 1: 3 in buffer, were applied, and the variation in absorbance was measured every 15 s for 120 s using a UV spectrophotometer (Istratoaie et al. 2022).

#### Determination of serum lipid profile

Serum total triglycerides, cholesterol, and high-density lipoprotein (HDL) were assessed using standard commercial kits (BioDiagnostics, Egypt), according to Allain et al. and Glick et al. (Allain et al. 1974; Glick et al. 1986).

#### Determination of serum NF-κB and IL-6

According to the manufacturing protocols, the serum NF-κB and IL-6 levels were quantified by an enzyme-linked immunosorbent assay (ELISA) (Frégeau et al. 2020; Huynh et al. 2018).

### Total RNA isolation and quantitative real-time PCR (Q-RT-PCR) assay

Total RNA was extracted from the heart tissues of all groups using the TriZol (Invitrogen, CA, USA) and chloroform techniques. After dissolving the samples in water free of RNase, the final concentrations were adjusted to 100 ng/mL. Then, 10 µL of each pure RNA sample were used to generate a cDNA using a commercial kit (Qiagen, MD, USA). Total RNA was incubated with reverse transcriptase and oligo-(dT) primers at 45 °C for an hour and then at 95 °C for five minutes, as instructed by the manufacturer. cDNA was kept at 20 °C until analysis.

The expression levels of several genes linked to hypertension in cardiac tissues were estimated using real-time qPCR. As stated in (Table 2), cDNA was used as a template for PCR amplification using DNA polymerase and specific primers. The relative gene expressions were determined using the Quanti-Tect SYBR Green PCR Kit (Qiagen). Primer Express software (version 2.0) generated oligonucleotides specific to AT1, AT2, ACE1, ACE2, Mas1l, NRF2, NF-κB, GAPDH, and actin genes. Using an Applied Bio-system device, the real-time PCR reaction was carried out at 50 °C for 2 min and 95 °C for 10 min. An additional 40 cycles of 95 °C for 30 s, annealing at 60 °C for 30 s, and extension at 72 °C for 30 s were then performed. The threshold cycle (CT) results were analyzed utilizing SDS 2.2.

**Table 2:** qPCR murine primer sequences

Gene	Type	Primers	NCBI gene ID
AT1 (Agtr1a)	F1 R1	5′-TCGGCCAAAAGCCTGCGTCT-3′ 5′-GGCAGGGTGAATGGTCCTTTGGT-3′	24,180
AT2 (Agtr2)	F1 R1	5′-ATCTGGCTGTGGCTGACTTACTCCT-3′ 5′-GCACATCACAGGTCCAAAGAGCCAG-3′	24,182
ACE	F1 R1	5′-ACCGCCGCTATGGGGACAAATACA-3′ 5′-AAATGTTCTCCCAGCTCTGCGCC-3′	24,310
ACE2	F1 R1	5′-AAGTGGTGGGAGATGAAGCGGGA-3′ 5′-ATGGAACAGAGATGCAGGGTCACAGT-3′	302,668
Mas1l	F1 R1	5′-GCCCCAGTTCCCAGAATGCCGATA-3′ 5′-GCTGGTTGACGTGGCTTCAGGTT-3′	404,641
GAPDH	F1 R1	5′-TTGTGCAGTGCCAGCCTCGT-3′ 5′-TCACAAGAGAAGGCAGCCCTGGT-3′	24,383
NRF2 (Nfe2l2)	F1 R1	5′-CCCAGGTTGCCCACATTCCCAAAC-3′ 5′-GAATATCCAGGGCAAGCGACTCATGG-3′	83,619
NF-KB	F1 R1	5′-TGGAGCAAGCCATTAGCCAGCG-3′ 5′-CCGCATTCAAGTCATAGTCCCCGC-3′	309,165

### Western immunoblotting

Isolated cardiac tissues were lysed in ice-cold lysis buffer containing 1% Triton X-100 and 10 mM sodium pyrophosphate, supplemented with protease inhibitor. Protein concentrations were quantified by Bradford assays. Lysate proteins were solubilized in Laemmli buffer and subjected to SDS–PAGE for separation (Elimam et al. 2019). Proteins were electrophoretically transferred to polyvinylidene difluoride membranes. Membranes were incubated at 22 °C for one hr with 5% BSA in 1 × Tris-buffered saline containing 0.1% Tween 20. The membranes were incubated overnight with primary antibodies in a 5% BSA. This was followed by a 1-h incubation at a temperature of 22 °C with the secondary antibody called goat anti-rabbit IgG, which was coupled with horseradish peroxidase. Membranes were developed using ECL (Elimam et al. 2013; Elimam et al. 2015). The quantitative densitometry of protein bands was conducted using ImageJ software (National Institutes of Health, Bethesda, MD).

### Statistical analysis

One-way ANOVA was employed to analyze the data (mean ± SE). Where significant differences were identified, individual comparisons were conducted between groups utilizing Holm-Sidak’s multiple comparisons test (post hoc analysis) (GraphPad Prism® version 9.00, San Diego, CA, USA). Statistical significance was defined as a probability value of < 0.05.

## Results

### Formulation and characterization of VST- and HCTZ-loaded SNEDS

**Fig. 1:**
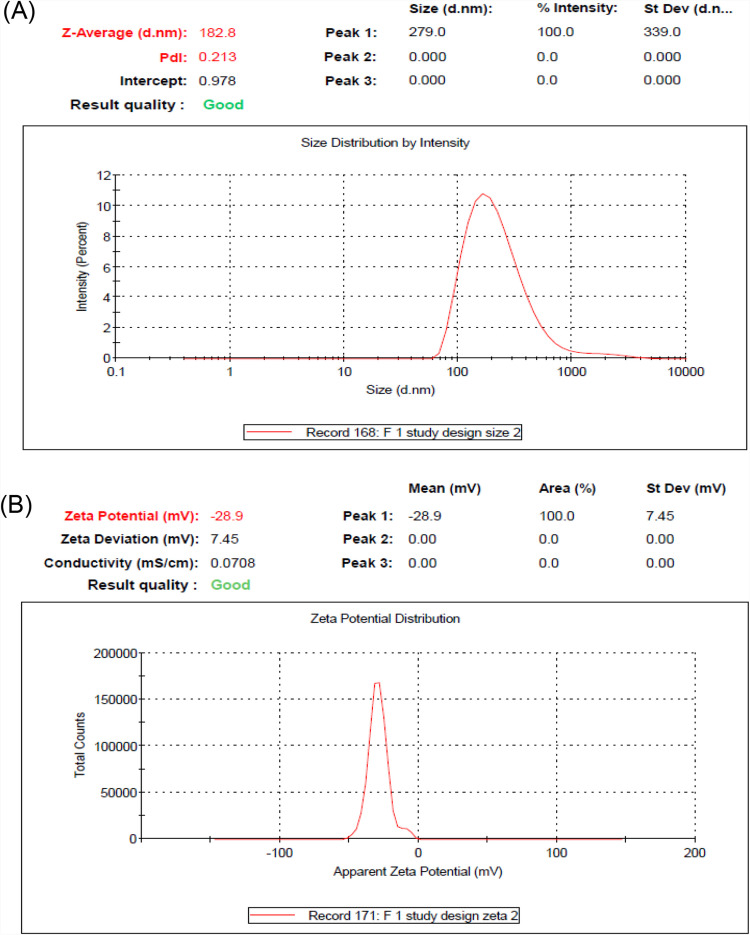
Characterization of SNEDS-loaded VST and SNEDS-loaded VST/HCTZ: **A** particle size and **B** zeta potential

The VST- and HCTZ-loaded SNEDS were successfully formulated with a composition of 24.9% sesame oil, 33.3% Tween 80, and 41.8% PEG 400. The formulations were optimized for minimal globule size and maximum drug loading capacity. The visual evaluation confirmed that the SNEDS had a uniform appearance and a high tendency for spontaneous emulsification. Phase separation and thermodynamic stability tests, including freeze–thaw cycles and centrifugation, indicated stable formulations without creaming or cracking. The particle size, polydispersity index (PDI), and zeta potential were 182.8 nm, 0.213, and − 28.9 mV, respectively. These findings revealed that the formulations had a consistent particle size distribution and stability, as displayed in Fig. 1a, b.

### Evaluation of the prepared tablet formulations

As shown in Table 3, LST-1 and LST-2 exhibited HR values of 1.23 and 1.21 and CI values of 18.42% and 17.50%, respectively, indicating fair flow properties. Meanwhile, DCT-3 and DCT-4 showed better flow properties with HR values of 1.15 and 1.14 and CI values of 13.33% and 12.00%, indicating good flow.

**Table 3:** Pre- and post-compression properties of the prepared tablet formulations

Formula code	Pre-compression properties			Post-compression properties					
	HR^a^	CI (%)^a^	Type of flow	Weight (mg)^c^	Friability (%)^c^	Hardness (N)^b^	Disintegration time (min)^b^	VST Content (%)^c^	HCTZ content (%)^c^
LST-1	1.23	18.42	Fair	286.4	0.11	47.5	6.42	96.2	0
LST-2	1.21	17.50	Fair	289.525	0.16	46.2	4.81	95.9	96.6
DCT-3	1.15	13.33	Good	186.4	0.09	56.6	10.61	100.3	0
DCT-4	1.14	12.00	Good	189.525	0.06	62.1	13.47	97.7	98.9

*HR* Hausner ratio, *SCS* specific crushing strength, *HFR* hardness friability ratio

Types of flow according to Ph. Eur. 2.9.36

Note: the data are expressed as mean, and the standard deviation is presented in parentheses

^a^*n* = 3

^b^*n* = 6

^c^*n* = 10

LST formulations (LST-1 and LST-2) had weights around 286.4 mg and 289.525 mg, with friability of 0.11% and 0.16%, respectively, indicating good mechanical strength. Meanwhile, DCT formulations (DCT-3 and DCT-4) were lighter at 186.4 mg and 189.525 mg, with even lower friability of 0.09% and 0.06%, showing excellent mechanical integrity.

LST-1 and LST-2 had hardness values of 47.5 N and 46.2 N, respectively, indicating adequate tablet strength. On the other hand, DCT-3 and DCT-4 demonstrated higher hardness values of 56.6 N and 62.1 N, reflecting superior mechanical strength.

LST-1 and LST-2 disintegrated in 6.42 min and 4.81 min, respectively, showing relatively rapid disintegration. DCT-3 and DCT-4 had longer disintegration times of 10.61 min and 13.47 min, indicating slower disintegration rates.

VST content was consistent across all formulations, with LST-1 and LST-2 showing 96.2% and 95.9%, and DCT-3 and DCT-4 showing 100.3% and 97.7%, respectively. HCTZ content in LST-2 and DCT-4 was also uniform, at 96.6% and 98.9%, respectively.

Complete pre-formulation, formulation, and optimization of the nanoformulations were performed previously by implementing the quality-by-design approach (simplex-lattice mixture design) (El-Say et al. 2023) (Table 3).

### Effect of VST and VST/HCTZ liquisolid tablets on pulse rate and the mean arterial blood pressure in L-NAME-induced hypertensive rats

**Fig. 2:**
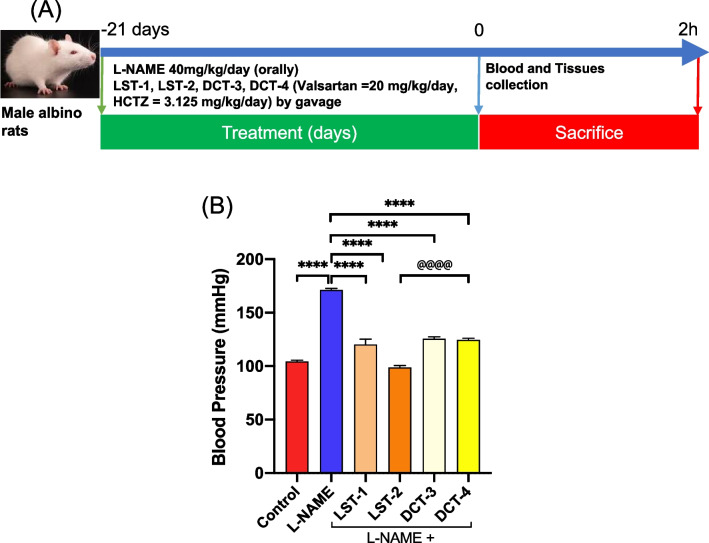
SNEDS-loaded VST and SNEDS-loaded VST/HCTZ reduce blood pressure: **A** study design. **B** Bar graph represents the blood pressure. The administration of formulations LST-1, LST-2, DCT-3, or DCT-4 substantially reduces blood pressure. LST-2 reduced BP significantly compared to DCT-4. ^****^P < 0.0001, ^@@@@^*P* < 0.0001 (ANOVA)

L-NAME administration resulted in a significant increase in mean arterial blood pressure (MAP) and pulse rate compared with the normal control rats, indicating the induction of hypertension. Figure 2B illustrates that rats treated with L-NAME (40 mg/kg/day) had a 38% increase in MAP compared to normal control rats after three weeks. Likewise, the heart rate of the rats fed L-NAME fell by 20% after three weeks (Table 3). The administration of formulations LST-1, LST-2, DCT-3, or DCT-4 substantially reduced MAP and pulse rate in hypertensive rats. Interestingly, the SNEDS-loaded VST/HCTZ formulation, LST-2, decreased the BP significantly compared to DCT-4 (Fig. 2B).

### Effect of VST and VST/HCTZ liquisolid tablets on ECG in L-NAME-induced hypertensive rats

L-NAME augmented the R-R, QT, and PR intervals and R-amplitude. On the other hand, it reduced heart rate and QRS compared to normal control rats. Notably, LST-1, LST-2, DCT-3, and DCT-4 improved the L-NAME-induced alterations in ECG parameters after 21 days of treatment (Table 4 and Fig. 11).

**Table 4:** Effect of VST and VST/HCTZ liquisolid tablets on ECG parameters

	Control	L-NAME	L-NAME + LST-1	L-NAME + LST-2	L-NAME + DCT-3	L-NAME + DCT-4
RR interval (s)	0.1393 ± 0.00004	0.1732 ± 0.0004^*^	0.1606 ± 0.00099^*^	0.1395 ± 0.00007^@^	0.1562 ± 0.00048^*@^	0.173 ± 0.00226^*^
Heart rate (BPM)	430.9 ± 0.1626	346.4 ± 0.8037^*^	369.3 ± 2.076^*^	430.2 ± 0.2394^@^	384.2 ± 1.178^*@^	347.3 ± 4.383^*^
PR interval (s)	0.0486 ± 0.00003	0.0539 ± 0.00015^*^	0.049 ± 0.00097^@^	0.0371 ± 0.00051^*@^	0.0426 ± 0.00021^*@^	0.0589 ± 0.0001^@^
QRS interval (s)	0.016 ± 0.00005	0.0134 ± 0.00011^*^	0.0166 ± 0.00018^@^	0.0165 ± 0.00017^@^	0.0167 ± 0.00015^@^	0.0169 ± 0.0005^@^
QT interval (s)	0.0377 ± 0.0001	0.0545 ± 0.00241^*^	0.0516 ± 0.00187^=*^	0.0532 ± 0.00051^*^	0.0465 ± 0.00021^*@^	0.039 ± 0.001^@^
R amplitude (mV)	0.3681 ± 0.00119	0.5666 ± 0.00546^*^	0.4736 ± 0.01244^*@^	0.3476 ± 0.00098^@^	0.5428 ± 0.00187^*^	0.337 ± 0.01554^@^

Data were expressed as mean ± SE. of 3–5 rats per group

^*^vs. control group

^@^vs. L-NAME group

### Effects of VST and VST/HCTZ liquisolid tablets on histopathology of the heart in L-NAME-induced hypertensive rats

**Fig. 3:**
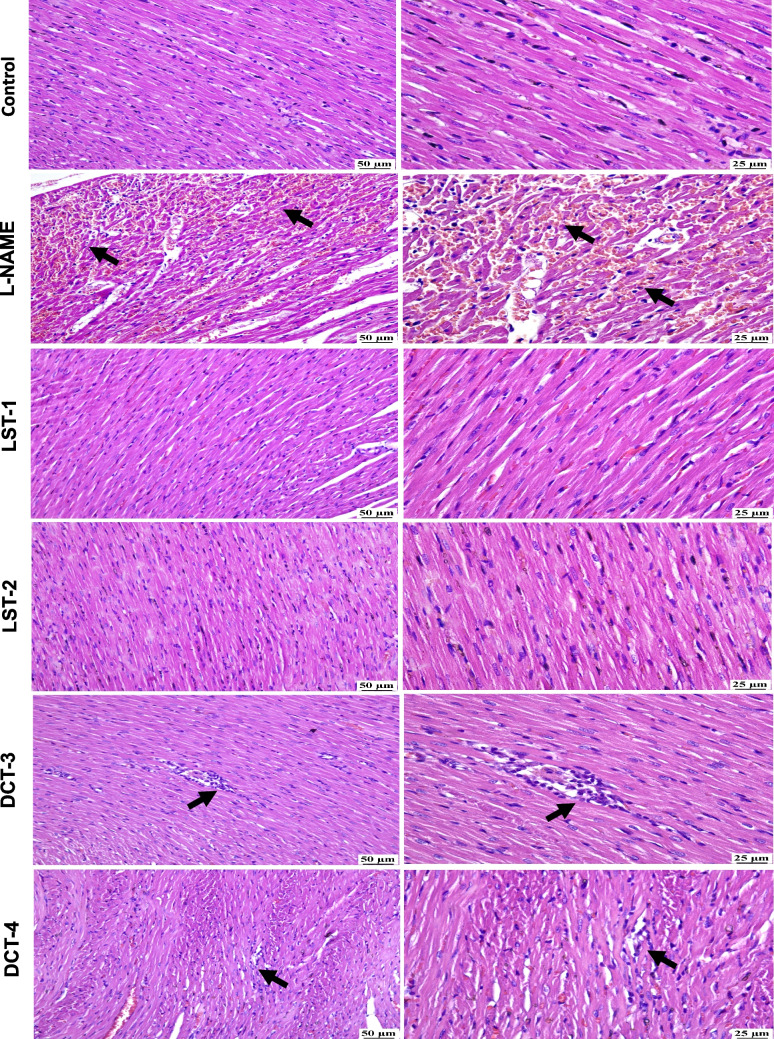
Histopathology of the heart in L-NAME-induced hypertensive rats. Microscopic examination of hearts from the control group showed normal myocardium without any detectable alterations. L-NAME-induced hypertension group revealed diffuse myocarditis manifested by mononuclear inflammatory cell infiltration (black arrow). Normal myocardium was detected in LST-1 and LST-2. Mild mononuclear inflammatory cell infiltration in between myocardial fibers and small focal aggregation of mononuclear inflammatory cells were shown in DCT-3 and DCT-4, respectively (black arrows)

Microscopic examination of hearts from the control group showed normal myocardium without any detectable alterations (Fig. 3). The L-NAME-induced hypertension group revealed diffuse myocarditis manifested by mononuclear inflammatory cell infiltration between muscle bundles with intermuscular hemorrhage in some of the examined sections (Fig. 3). Administration of formulation LST-1 (SNEDS-loaded VST LSTs) showed normal myocardium (Fig. 3); likewise, a normal myocardium was detected in the LST-2 (SNEDS-loaded VST/HCTZ LSTs) group (Fig. 3). DCT-3 (non-SNEDS-loaded VST-DCTs) group (Fig. 3) exhibited mild mononuclear inflammatory cell infiltration between myocardial fibers and perivascular lymphocytic infiltration. The DCT-4 (non-SNEDS-loaded VST/HCTZ-DCTs) group (Fig. 3) showed small focal aggregation of mononuclear inflammatory cells within the myocardium, although some other sections were normal.

### Effects of VST and VST/HCTZ liquisolid tablets on cardiac RAS mRNA expressions

**Fig. 4:**
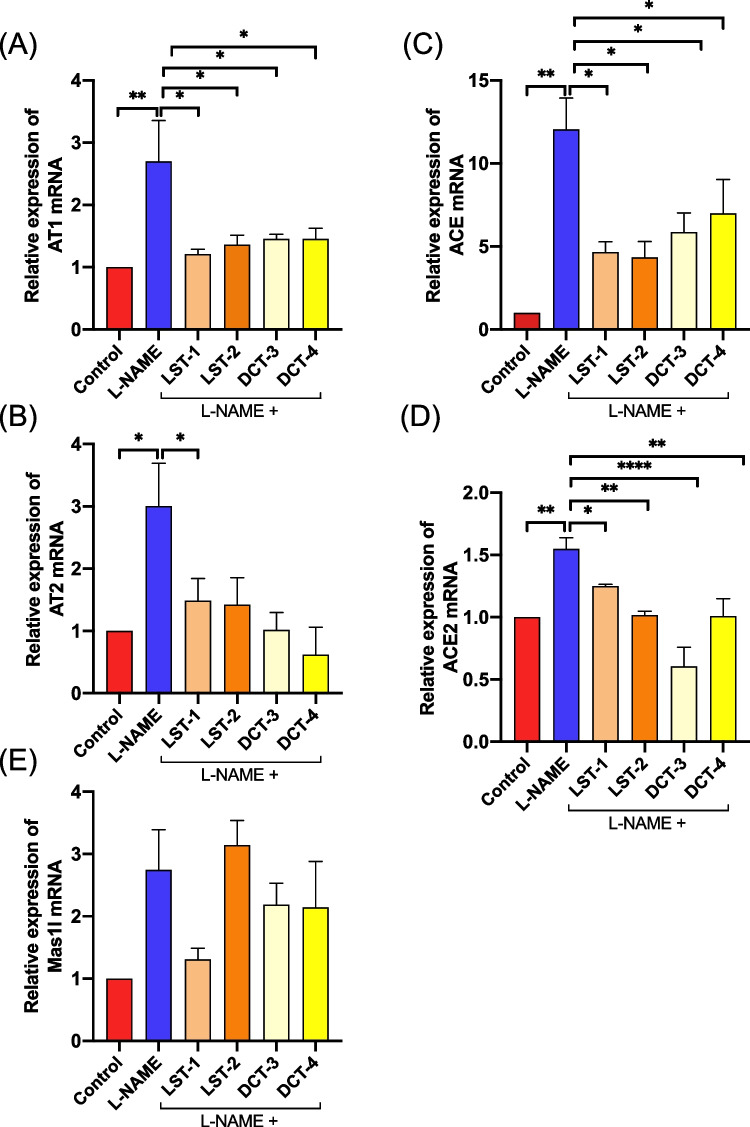
Cardiac RAS mRNA expressions: L-NAME upregulated the mRNA expressions of **A** AT1 R, **B** AT2 R, **C** ACE, and **D** ACE2. The administration of formulations LST-1, LST-2, DCT-3, or DCT-4 reduced the expression of AT1 R, AT2 R, ACE, and ACE2. **E** There are no significant differences in the Mas1l mRNA expression among groups. There are 6–8 rats per group. ^*^P < 0.05, ^**^P < 0.01, ^****^P < 0.0001 (ANOVA)

The mRNA expression of RAS components (ACE1, ACE2, AT1 R, AT2 R, and Mas1l) was analyzed with SYBR Green RT-PCR. GAPDH was used as an internal control for the mRNAs. As shown in Fig. 4A, C, the AT1 R and ACE mRNA expressions were upregulated in the L-NAME-treated rats, and administration of LST-1, LST-2, DCT-3, or DCT-4 formulations downregulated the AT1 R and ACE mRNA levels significantly. However, no significant differences were found between VST liquisolid tablets and VAS/HCTZ-liquisolid tablets (Fig. 4A, C). Likewise, the mRNA expressions of AT2 R and ACE2 (Fig. 4B, D) were significantly lower in LST-1, LST-2, DCT-3, and DCT-4 compared to L-NAME. However, Mas1l was not altered by the administration of any of the liquisolid tablet formulations (Fig. 4E).

### Effect of VST and VST/HCTZ liquisolid tablets on oxidative stress in L-NAME-induced hypertensive rats

**Fig. 5:**
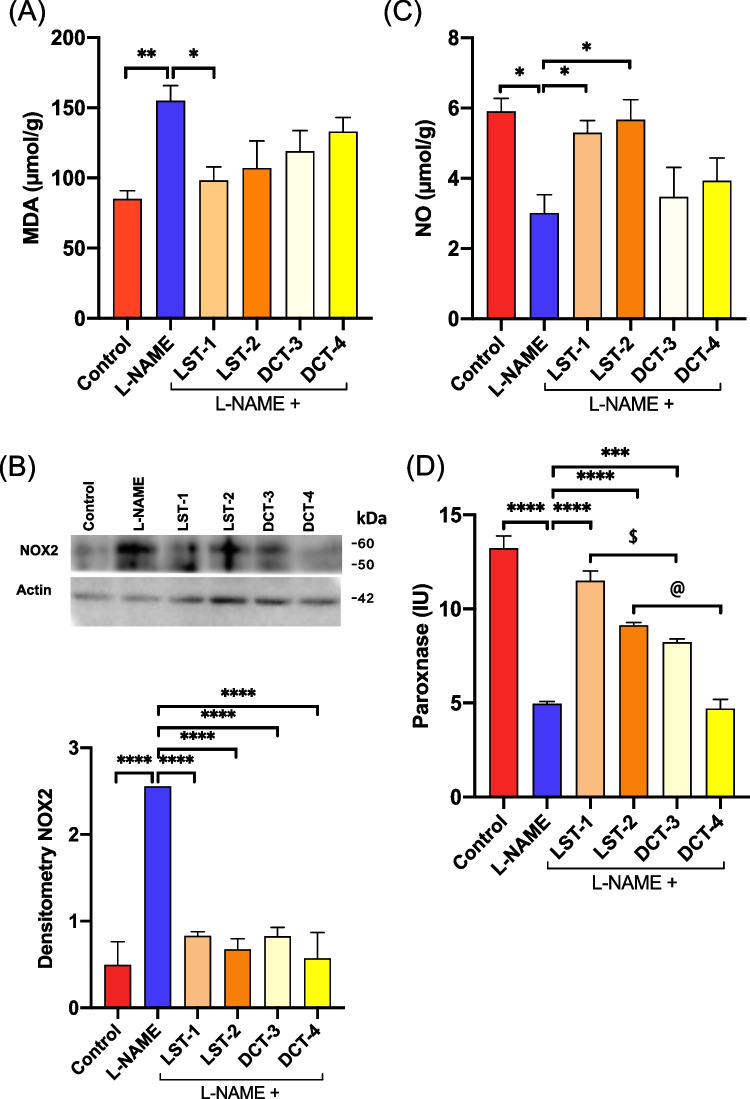
Oxidative stress in L-NAME-induced hypertensive rats after administration of SNEDS-loaded VST and SNEDS-loaded VST/HCTZ. **A** MDA levels in the aorta tissue homogenates were increased in the L-NAME-treated group versus the control group. LST-1 administration reduced the elevated MDA level. **B** The protein expression of NOX2 was upregulated in the L-NAME-treated group versus the control group and downregulated in all formulations treated rats. Signals were quantified by densitometry. ^****^P < 0.0001 L-NAME-treated group versus control; ^****^P < 0.0001 LST-1-, LST-2-, DCT-3-, and DCT-4-treated groups compared to the L-NAME-treated group. **C** NO level in aorta tissue homogenates was depleted in the L-NAME-treated group versus the control group. LST-1- and LST-2-treated groups increased NO levels compared to the L-NAME-treated group. There are no significant differences in DCT-3- and DCT-4-treated groups compared to the L-NAME-treated group. ^*^P < 0.05 (ANOVA). **D** Paraoxonase serum level was significantly reduced in the L-NAME-treated group versus control. The LST-1-, LST-2-, and DCT-3-treated rats significantly increased paraoxonase serum levels compared to the L-NAME-treated group. ^***^P < 0.001, ^****^P < 0.0001, ^$^*P* < 0.05 LST-1 versus DCT-3, ^@^*P* < 0.05 LST-2 versus DCT-4 (ANOVA). There are 6–8 mice per group

MDA level in the aorta was increased in the L-NAME-treated group. Only LST-1 significantly decreased MDA level compared to the L-NAME-treated group. LST-2, DCT-3, and DCT-4 tended to reduce the increase in MDA level by L-NAME (Fig. 5A). L-NAME-induced depletion of NO in the aorta tissue homogenates was associated with oxidative stress as shown in Fig. 5. LTS-1 and LTS-2 administration significantly increased the NO level versus L-NAME (Fig. 5C). Moreover, the antioxidant paraoxonase serum level was significantly reduced in the L-NAME-treated group by 61.5%, compared to those in control rats. The antioxidant defense was significantly increased in the LST-1-, LST-2-, and DCT-3-treated rats than in the L-NAME-treated rats by 135%, 90%, and 80%, respectively (Fig. 5D). Notably, the increase in paraoxonase activity in LST-1-treated rats was higher than in DCT-3, and LST-2-treated rats was higher than in DCT-4 (Fig. 5D).

#### Protein expression analyses

To investigate whether alterations at the functional levels correspond with the protein expression profile, we assessed the relative abundance of NADPH oxidase 2 (NOX2) (Elimam et al. 2024). NOX2 levels were elevated by fivefold in the heart tissue homogenate of hypertensive rats (L-NAME) compared to control rats. As shown in Fig. 5B, LTS-1, LTS-2, DCT-3, and DCT-4 significantly lowered the NOX2 expression in treated rats compared to the L-NAME group.

### Effects of VST and VST/HCTZ liquisolid tablets on lipid profile and liver markers in L-NAME-induced hypertensive rats

**Fig. 6:**
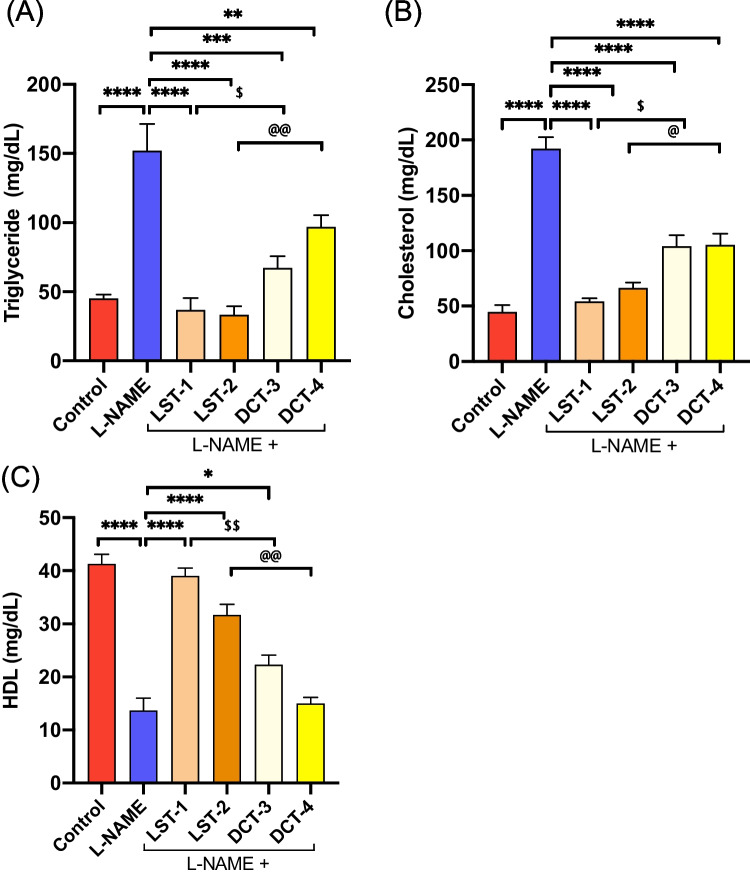
Lipid profile in L-NAME-induced hypertensive rats after SNEDS-loaded VST and SNEDS-loaded VST/HCTZ administration. **A** levels of triglycerides significantly decreased in LST-1-, LST-2-, DCT-3-, and DCT-4-treated groups compared to L-NAME-treated rats. ^**^P < 0.01, ^***^P < 0.001, ^****^P < 0.0001, ^@@^*P* < 0.01 LST-2 versus DCT-4. **B** Compared to L-NAME-treated rats, total cholesterol levels significantly decreased in LST-1-, LST-2-, DCT-3-, and DCT-4-treated groups. ^****^P < 0.0001, ^$^*P* < 0.01 LST-1 versus DCT-3, ^@^*P* < 0.01 LST-2 versus DCT-4. **C** HDL levels were increased in both formulations: LST-1 and LST-2. DCT-3 showed a moderate increase, and DCT-4 showed no change compared to L-NAME-treated rats. ^*^P < 0.05, ^****^P < 0.0001, ^$$^*P* < 0.01 LST-1 versus DCT-3, ^@@^*P* < 0.01 LST-2 versus DCT-4 (ANOVA). There are 6–8 mice per group

Dyslipidemia in L-NAME-hypertensive rats was confirmed by increased serum triglycerides and cholesterol and reduced HDL levels compared to the normal control rats (Fig. 6A–C). Administration of LST-1, LST-2, DCT-3, and DCT-4 tablets significantly altered the dyslipidemia and almost normalized the triglyceride and total cholesterol levels (Fig. 6A, B). In addition, LST-1, LST-2, and DCT-3 significantly increased the serum level of HDL versus L-NAME-treated rats (Fig. 6C).

**Fig. 7:**
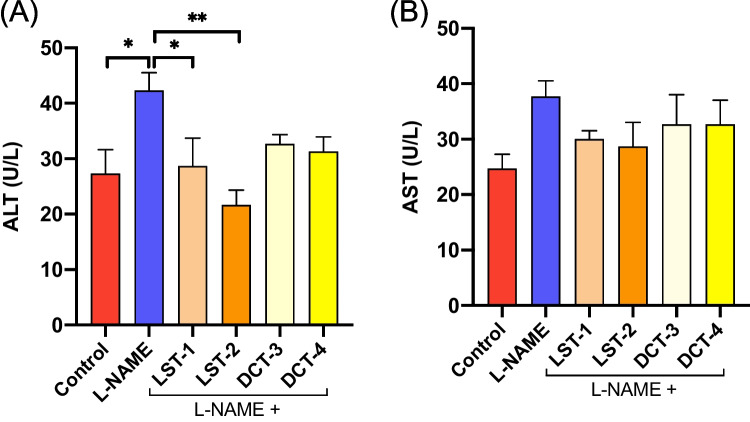
Liver marker enzyme activity (ALT and AST): (**A**) LST-1 and LST-2 lowered the ALT significantly versus L-NAME-induced hypertensive rats. *P < 0.05, **P < 0.01 (ANOVA). Both DCT-3 and DCT-4 tended to reduce the ALT. (**B**) All formulations did not significantly alter the AST level compared to the L-NAME-treated group. There are 6-8 mice per group

Prolonged suppression of nitric oxide synthase (NOS) by L-NAME modifies several biochemical indicators. L-NAME treatment resulted in a considerable elevation of liver marker enzyme activity (ALT and AST) compared to normal controls in the current study (Fig. 7A, B), indicating hepatic injury. The results suggest that LST-1 and LST-2 lowered the ALT significantly compared to L-NAME-induced hypertension in rats. Both DCT-3 and DCT-4 tended to reduce ALT. Noteworthy, all formulations did not significantly alter the AST level compared to the L-NAME-treated group (Fig. 7B).

### Impacts of VST and VST/HCTZ liquisolid tablets on the expressions of Nrf2 and PPAR genes in heart homogenate

**Fig. 8:**
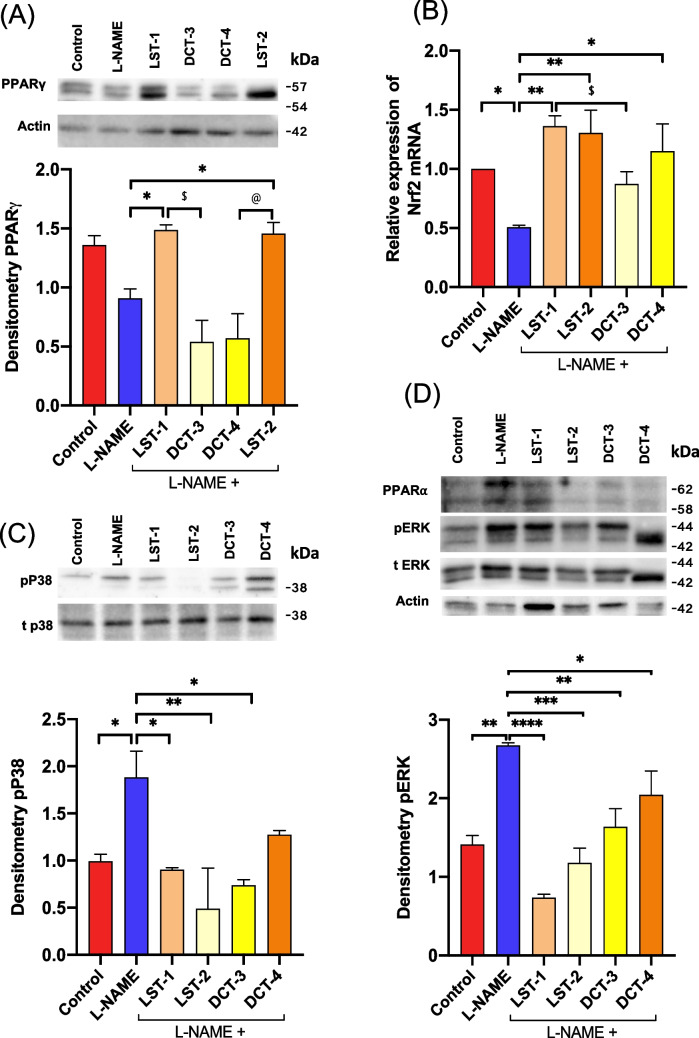
Downregulation of Nrf2 and PPARs gene expressions in L-NAME-induced hypertensive rats. **A** The protein expression of PPARγ was downregulated in the L-NAME-treated group versus the control group and upregulated in LST-1 and LST-2 formulations-treated rats. Signals were quantified by densitometry. ^*^P < 0.05 LST-1 versus L-NAME-treated group and LST-2 versus L-NAME-treated group; ^$^*P* < 0.05 LST-1 compared to DCT-3; ^@^*P* < 0.05 LST-2 compared to DCT-4-treated group. **B** mRNA expression of Nrf2 was significantly downregulated in the L-NAME-treated group versus the control group and upregulated in LST-1 and LST-2 formulations-treated rats. ^*^P < 0.05 L-NAME-treated group versus control; ^**^P < 0.01 LST-1 versus L-NAME-treated group and LST-2 versus L-NAME-treated group; ^*^P < 0.05 DCT-4 versus L-NAME-treated group; ^$^*P* < 0.05 LST-1 compared to DCT-3 (ANOVA). **C** The protein expression of pP38 was upregulated in the L-NAME-treated group versus the control group and significantly downregulated in LST-1-, LST-2-, and DCT-3-treated rats. Signals were quantified by densitometry. ^*^P < 0.05 L-NAME-treated group versus control and LST-1 versus L-NAME-treated group; ^**^P < 0.01 LST-1 compared to L-NAME-treated group; ^*^P < 0.05 DCT-3 compared to L-NAME-treated group. **D** The protein expressions of pERK (p42/44) and PPARα were upregulated in the L-NAME-treated group versus the control group and downregulated in all formulations-treated rats. Signals for pERK (p42/44) expressions were quantified by densitometry. ^**^P < 0.01 L-NAME-treated group versus control and DCT-3 versus L-NAME-treated group; ^****^P < 0.0001 LST-1 compared to L-NAME-treated group; ^***^P < 0.001 LST-2 compared to L-NAME-treated group; ^*^P < 0.05 DCT-4 compared to L-NAME-treated group. There are 6–8 mice per group

To further explain the effect of VST and VST/HCTZ liquisolid tablets on the antioxidant status in heart tissues, the relative gene expression and protein level of Nrf2 were measured using RT-qPCR. In L-NAME-treated rats, the relative gene expression of Nrf2 was reduced compared to control rats (Fig. 8B). The reduction of Nrf2 level was significant in L-NAME-treated rats compared to control; treatment of the rats with LST-1 or LST-2 showed a marked increase in the relative gene expression of Nrf2 compared to L-NAME-treated rats**.** Interestingly, the increase in Nrf2 expression following treatment of the rats with LST-1 was significantly different than the group treated with DCT-3 (Fig. 8B).

To investigate the mechanism of action of VST and VST/HCTZ liquisolid tablets in improving the reduction of BP, we studied one of the genes involved in hypertension, PPARs. Among the three PPAR isoforms, PPARγ has the most substantial genetic evidence as a principal regulator of BP. It was identified as a locus that interacts at the chromatin level, serving as a distal-related gene linked to BP features in a study that included one million individuals (Evangelou et al. 2018; Fang et al. 2021). Therefore, we undertook to measure the expression level of PPARγ in our study. The results show that LST-1 and LST-2 increased the PPARγ protein levels significantly compared to control and L-NAME-induced hypertension in rats (Fig. 8A). In addition, MAPKs such as p38 and ERK were phosphorylated (activated) in L-NAME-treated animals, and both VST and VST/HCTZ liquisolid tablet treatments reduced their activation. The reduction of pP38 and pERK was more profound by LST-1 and LST-2 treatments (Fig. 8C, D).

### The effect of VST and VST/HCTZ liquisolid tablets on the heart immunohistochemical localization of iNOS

**Fig. 9:**
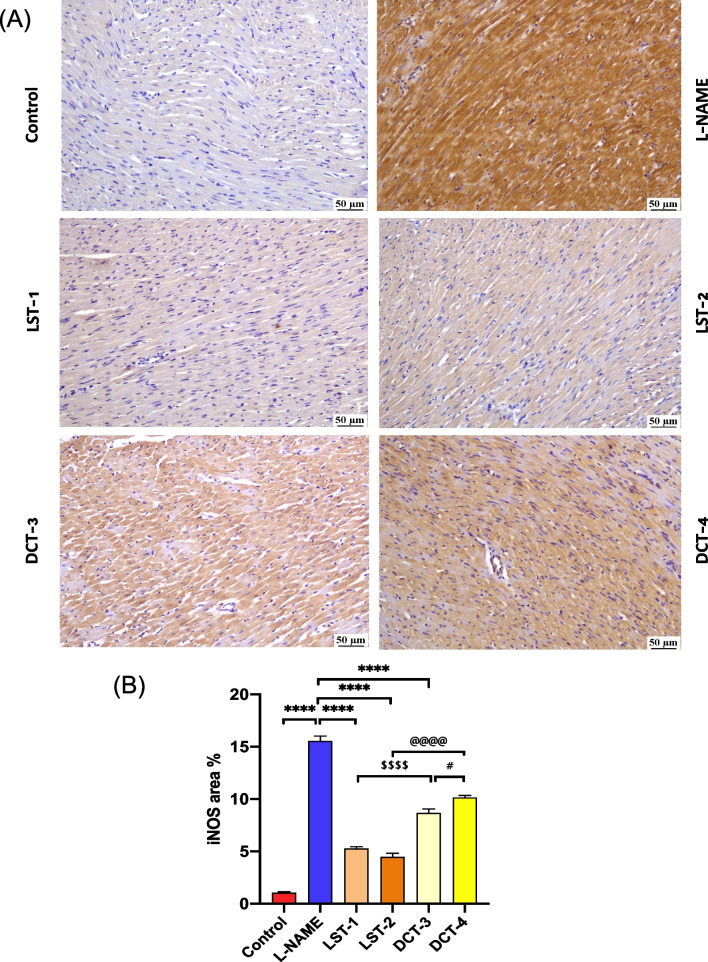
Photomicrographs of the heart immunostained with iNOS (× 200). **A** The iNOS immunoreactivity was characteristically cytoplasmic, and the cytoplasm was stained brown; the control normotensive group had minimal immunoreactivity. L-NAME group shows a very strong immunopositive reaction. LST-1 and LST-2 show weak immunoreactivity. DCT-3 and DCT-4 groups showed moderate levels of immunoreactivity. **B** The bar chart represents the iNOS immunopositivity expressed as area %. Mean values with different superscripts are significantly different. ^****^P < 0.0001 L-NAME-treated group versus control. ^****^P < 0.0001 LST-1, LST-2, and DCT-3 versus L-NAME-treated group; ^$$$$^*P* < 0.0001 LST-1 compared to DCT-3; ^@@@@^*P* < 0.0001 LST-2 compared to the DCT-4-treated group; ^#^*P* < 0.05 DCT-3 compared to the DCT-4-treated group (ANOVA)

To localize changes in NOS levels after L-NAME-induced hypertension, we performed immunohistochemical staining for iNOS in the heart from control, L-NAME, LST-1, LST-2, DCT-3, and DCT-4. Examination of cardiac myocytes revealed a significantly higher expression of iNOS in L-NAME-treated rats compared to the other experimental groups. Moderate iNOS expression was recorded in the DCT-3 and DCT-4 groups. The absence of a significant difference was observed between the LST-1 and LST-2 groups. Samples incubated just with a secondary antibody exhibited no staining for iNOS (Fig. 9).

### The effect of VST and VST/HCTZ liquisolid tablets on inflammatory cytokines (NF‑κB and IL-6)

**Fig. 10:**
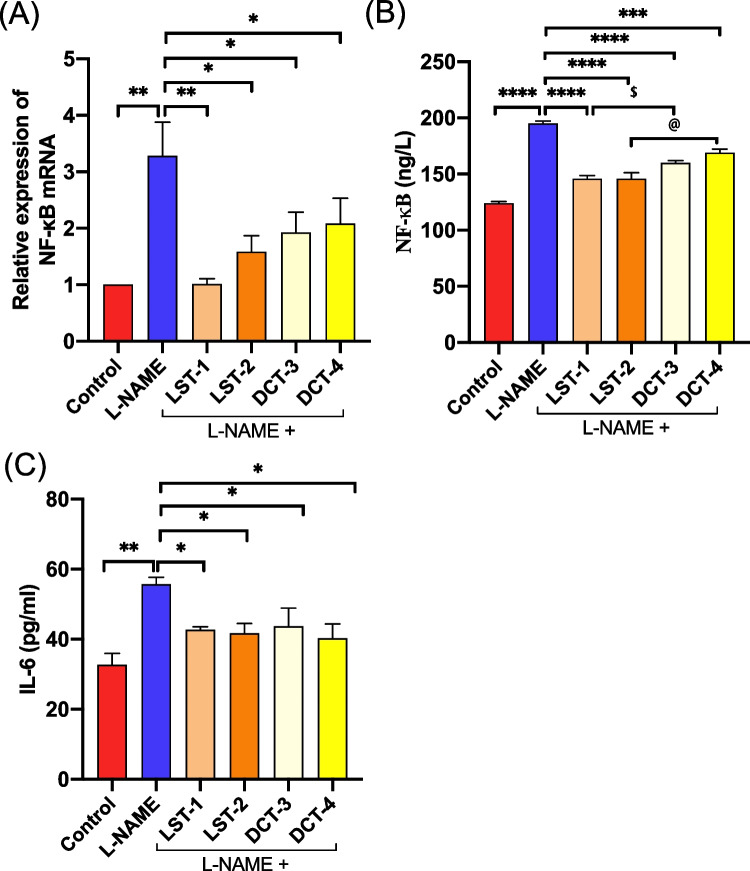
Inflammatory cytokines in L-NAME-induced hypertensive rats after SNEDS-loaded VST and SNEDS-loaded VST/HCTZ administration. **A** mRNA expression of NF-κB was increased in heart tissues of the L-NAME-treated group. All formulations reduced the NF-κB mRNA significantly. ^**^P < 0.01 L-NAME-treated group versus control and LST-1 versus L-NAME-treated group; ^*^P < 0.05 LST-2, DCT-3, and DCT-4 versus L-NAME-treated group. **B** Serum NF-κB level also increased in the L-NAME-treated group, and all formulations reduced its level. ^****^P < 0.0001 L-NAME-treated group versus control. ^****^P < 0.0001 LST-1, LST-2, DCT-3, and DCT-4 versus L-NAME-treated group; ^$^*P* < 0.05 LST-1 compared to DCT-3; ^@^*P* < 0.05 LST-2 compared to DCT-4-treated group. **C** Serum IL-6 level increased in the L-NAME-treated group, and all formulations reduced its level. ^**^P < 0.01 L-NAME-treated group versus control; ^*^P < 0.05 LST-1, LST-2, DCT-3, and DCT-4 compared to L-NAME-treated group. There are 6–8 mice per group

For an explanation of the mechanism of protection of LST-1 and LST-2 tablets against L-NAME-induced heart injury, the relative gene expression of NF-κB was assessed in the heart tissues using RT-qPCR, and in the serum, to assess the levels of NF-κB by ELISA. Rats treated with L-NAME only showed a threefold increase in gene expression and a twofold increase in the serum level of NF-κB compared to control rats (Fig. 10A, C, respectively). While the combination of L-NAME with LST-1, LST-2, DCT-3, or DCT-4 tablets caused almost 74%, 60%, 40%, and 30% decreases in the relative myocardial gene expression of NF-κB, respectively, with a consequent reduction in the serum levels by about 30%, 30%, 25%, and 20% (Fig. 10A, B, respectively). Along with reduced NF-κB signaling, gene expression of proinflammatory cytokine IL-6 was reduced in LST-1, LST-2–, DCT-3-, or DCT-4-treated rats (Fig. 10C).

## Discussion

The present study demonstrates the protective activities of VST and a combination of VST with HCTZ against cardiac stress caused by the L-NAME-induced hypertension model in rats by using different formulation preparations. VST and VST/HCTZ liquisolid tablets inhibit AT1 receptor gene expression (Fig. 4A) via activation of PPARγ and Nrf2 gene expression, as well as by inhibiting MAPK signaling pathways, including P38 and ERK kinases in the L-NAME-induced hypertension model. Activating the transcription factor Nrf2, which diminishes oxidative stress and inflammatory pathways, is essential to VST’s protective actions against hypertension. A significant mechanism elucidating the glucosylxanthone drug’s beneficial protective effect was evidenced by the activation of PPARγ, alongside the inhibition of pp38 MAPK and pERK, thus suppressing the pivotal transcription factor, NF-κB.

The development of hypertension is significantly influenced by oxidative stress. It is noteworthy that oxidative stress positively regulates AT1R function, a crucial factor in the etiology of hypertension. Antioxidant therapy has been demonstrated to lower BP, and the redox-sensitive transcription factor Nrf2 may hold key information about the antihypertensive properties of antioxidants (Barancik et al. 2016). Numerous genes encoding antioxidant proteins, such as glutathione peroxidase, hemoxygenase-1, and superoxide dismutase-2, are transcribed by Nrf2 binding to antioxidant response elements (Wang et al. 2018). The smallest gaseous intercellular signaling molecule, NO, mediates vascular relaxation and is less synthesized and metabolically active when NOS is inhibited (ref). Consequently, NO deficiency leads to systemic vasoconstriction and hypertension (Ahmad, et al. 2018). It has previously been demonstrated that low Nrf2 function in spontaneously hypertensive rats is linked to oxidative stress and vascular dysfunction because of an excessive buildup of Bach-1 in the arteries, which inhibits Nrf2 from initiating the antioxidant response elements (Lopes et al. 2015). Therefore, focusing on Nrf2 activation may be relevant because it can simultaneously decrease vascular ROS while increasing PPARγ and NO. As a result, it may be widely used to minimize the complications of hypertension. Here, in the present study, we examined how the induction of cardiac Nrf2 in response to VST as an antioxidant activates PPARγ (a pleiotropic nuclear receptor whose ligands reveal BP-reducing effects) (Fig. 8A) and thereby normalizes AT1R expression and reduces BP. According to these findings, Nrf2/PPARγ may have a role in BP regulation in a capacity other than through its antioxidant defense mechanism.

In addition, other evidence about PPAR isoforms is that activation of PPARα prevents or attenuates hypertension. For example, Diep et al. (Diep et al. 2002) showed that PPARα activation with docosahexaenoic acid inhibited Ang II-induced hypertension development in Sprague–Dawley rats. Another study offered similar evidence by showing that a combination of low doses of PPARα (fenofibrate) and PPARγ (rosiglitazone) activators reduced the hypertension development in the model of Ang II–infused Sprague–Dawley rats (Ciuceis et al. 2007). In the present study, SNEDS-loaded VST and SNEDS-loaded VST/HCTZ were shown to increase the expression levels of PPARγ but not PPARα (Fig. 8A, D).

BP and vascular tone are maintained by NO, the smallest gaseous intercellular signaling molecule (vasodilator) generated by the endothelium (Furchgott and Zawadzki 1980). Previous studies have reported that NO-deficient hypertension is linked to left ventricular hypertrophy because it raises renin and testosterone levels, activates ACE, and increases the expression of ATI, which has a direct vasoconstrictive effect (Simko et al. 2018). Additionally, diminished cGMP-mediated inhibition of protein synthesis and vascular smooth muscle proliferation results in reduced responsiveness and structural remodeling in the aorta when NO production is reduced (Simko et al. 2018). The current work demonstrated that decreased ventricular hypertrophy was linked to LST-1 and LST-2 restoring NO levels in L-NAME-induced hypertensive rats (Fig. 5C), in addition to enhancing the aortic and heart lesions’ histology score.

**Fig. 11:**
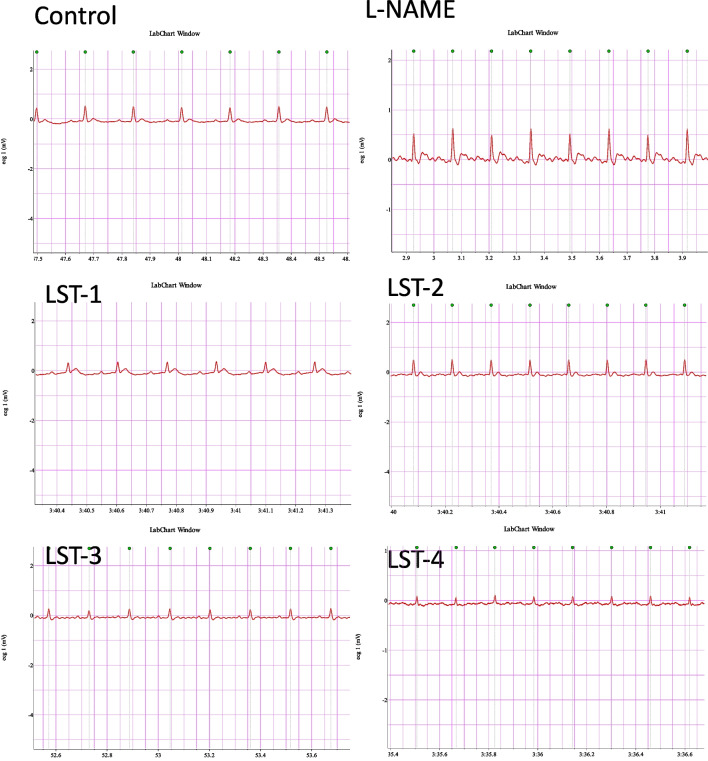
Electrocardiography (ECG): the ECG of rats was recorded for 5 min using the ECG PowerLab module. VST and VST/HCTZ ameliorated the L-NAME-induced changes in ECG parameters after 21 days of treatment

The R-amplitude, QT, PR, and R-R intervals were all increased by L-NAME (Table 2). Conversely, it decreased QRS and heart rate compared to normal control rats. The impairment of ECG parameters by L-NAME is in agreement with Chaswal et al.’s findings (Abdel-Rahman et al. 2017), which could be attributed to the decreased NO production of vascular smooth tone (Chaswal et al. 2012). After 21 days of treatment, it was noteworthy that all liquisolid VST and VST/HCTZ tablets reduced the L-NAME-induced alterations in ECG parameters (Table 2 and Fig. 11). Furthermore, because L-NAME suppresses NO, which disrupts sympathetic cardiovascular regulation, the observed elevated pulse rate in the L-NAME-induced hypertensive rats may be a factor in vascular remodeling and the development of cerebral hypertrophy (Regrigny et al. 2000).

The LST formulations demonstrated fair pre-compression flow properties, adequate post-compression mechanical strength, and rapid disintegration times. In contrast, the DCT formulations exhibited better flow properties and higher mechanical strength but slower disintegration times. Both types maintained consistent drug content uniformity, underscoring the effectiveness of the SNEDS approach in enhancing the dissolution and stability of VST and HCTZ. They will most likely become accessible to the pleiotropic nuclear receptor, whose ligands exhibit BP-reducing effects.

## Conclusion

The current investigation demonstrates the remarkable antihypertensive potential of the novel formula, “SNEDS-loaded VST and SNEDS-loaded VST/HCTZ.” After three weeks of treatment, the increased blood pressure and pulse rate were markedly normalized by the VST/HCTZ combination injected into SNEDS. Additionally, the dyslipidemic impact of L-NAME-induced hypertension was effectively reversed by SNEDS-loaded VST/HCTZ. Furthermore, the current work proposed a multimechanistic mechanism that mediates the antihypertensive effect of SNEDS-loaded VST and SNEDS-loaded VST/HCTZ. This mechanism includes the reduction of iNOS expression, antioxidant activity, and AT1R normalization action through activation of the Nrf2/PPARγ signaling cascade. These results highlight the increased advantages of using VST loaded with SNEDS or VST/HCTZ in combination as an adjuvant treatment for hypertension and associated consequences.

## Data Availability

All source data for this work (or generated in this study) are available upon reasonable request.
